# Family Solidarity, Social Support, Loneliness, and Well-Being Among Older Adults in Rural China

**DOI:** 10.1093/geroni/igaa057.1023

**Published:** 2020-12-16

**Authors:** Xiaoyan Zhang, Merril Silverstein

**Affiliations:** Syracuse University, Syracuse, New York, United States

## Abstract

China is experiencing a large increase in elderly population. In 2019, China’s population aged 60 and above had reached 253 million, accounting for 18.1% of the total population (National Bureau of Statistics of China, 2020). By 2050, the number of adults aged 60+ would be up to 430 million, reaching one third of the total population (Du, Zhai & Chen, 2005). Considering such a rapid aging process and the existing large number of older adults in China, it becomes imperative to investigate how psychosocial factors affect this group’s subjective well-being. This study proposed that, among older adults, higher support received from each of the three relational sources (adult children, family and friends) were associated with reduced loneliness and improved well-being. Structural equation modeling was conducted using a sample of rural adults aged 60 and older (N= 1142) from the 2018 wave of data from the Longitudinal Study of Older Adults in Anhui Province, China. Findings indicated that support from adult children directly and indirectly decreased older adults’ depression and improved their life satisfaction through loneliness; while support from family members directly decreased depression but did not directly improve life satisfaction or indirectly improve well-being through loneliness. Although support from friends did not have a significant impact on older adults’ well-being, it indirectly improved well-being through reduced loneliness. Findings have implications for programs or interventions targeting both parent -adult-child support and friends support and reducing rural older adults’ loneliness.

